# The Interaction of Arbuscular Mycorrhizal Fungi and Phosphorus Inputs on Selenium Uptake by Alfalfa (*Medicago sativa* L.) and Selenium Fraction Transformation in Soil

**DOI:** 10.3389/fpls.2020.00966

**Published:** 2020-06-26

**Authors:** Qi Peng, Miaomiao Wu, Zekun Zhang, Rui Su, Honghua He, Xingchang Zhang

**Affiliations:** ^1^ State Key Laboratory of Soil Erosion and Dryland Farming on the Loess Plateau, Northwest A&F University, Yangling, China; ^2^ Institute of Soil and Water Conservation, Chinese Academy of Sciences and Ministry of Water Resources, Yangling, China; ^3^ University of Chinese Academy of Sciences, Beijing, China; ^4^ College of Natural Resources and Environment, Northwest A&F University, Yangling, China

**Keywords:** alkaline phosphatase activity, *Glomus mosseae*, legume, phosphorus, rhizosphere carboxylates, selenium fraction

## Abstract

Selenium (Se) is a beneficial element to plants and an essential element to humans. Colonization by arbuscular mycorrhizal fungi (AMF) and supply of phosphorus (P) fertilizer may affect the bioavailability of Se in soils and the absorption of Se by plants. To investigate the interaction between AMF and P fertilizer on the transformation of soil Se fractions and the availability of Se in the rhizosphere of alfalfa, we conducted a pot experiment to grow alfalfa in a loessial soil with three P levels (0, 5, and 20 mg kg^-1^) and two mycorrhizal inoculation treatments (without mycorrhizal inoculation [−AMF] and with mycorrhizal inoculation [+AMF]), and the interaction between the two factors was estimated with two-way ANOVA. The soil in all pots was supplied with Se (Na_2_SeO_3_) at 1 mg kg^-1^. In our results, shoot Se concentration decreased, but plant Se content increased significantly as P level increased and had a significant positive correlation with AMF colonization rate. The amount of total carboxylates in the rhizosphere was strongly affected by AMF. The amounts of rhizosphere carboxylates and alkaline phosphatase activity in the +AMF and 0P treatments were significantly higher than those in other treatments. The concentration of exchangeable-Se in rhizosphere soil had a positive correlation with carboxylates. We speculated that rhizosphere carboxylates promoted the transformation of stable Se (iron oxide-bound Se) into available Se forms, i.e. exchangeable Se and soluble Se. Colonization by AMF and low P availability stimulated alfalfa roots to release more carboxylates and alkaline phosphatase. AMF and P fertilizer affected the transformation of soil Se fractions in the rhizosphere of alfalfa.

## Highlights

1. AM inoculation and P input enhanced alfalfa P and Se accumulation and availability
2. Carboxylates and alkaline phosphatase affected Se translocation in soil-alfalfa systems
3. AM inoculation and P input increased Se availability through increasing EX-Se
4. Fe-Se and EX-Se could reflect Se availability in loessial soil

## Introduction

Selenium (Se) is a naturally occurring metalloid element which is essential at low concentrations to humans and animals, but toxic at high concentrations (Sors et al., 2005). Se concentrations in staple foods should not be lower than the critical standard of 100 μg kg^-1^, below which a human’s basic needs cannot be met (Combs, 2001). Low intakes of Se by humans can cause health disorders and increase the risk of cancers (Liu et al., 2004; Whanger, 2004; He et al., 2017a). Se in soil can enter food chain *via* plant uptake, and humans mainly acquire dietary Se from plant-based foods through the food chain (Rayman, 2000; Yu et al., 2011). Besides, low concentrations of Se in soil can upregulate the production of higher plant enzymes (e.g., peroxidases and reductases) and protect plants from abiotic stresses (Wang et al., 2019), and have beneficial effects on plant growth and yield (Wang, Q. et al., 2016; White, 2018). Therefore, in agricultural production, the application of Se fertilizers is one of the effective ways to increase plant Se uptake, thus meeting the requirements of human health (Zhang et al., 2014).

The bioavailability of Se in soils is not only affected by soil properties such as pH, redox conditions, organic matter content, and synergy or antagonism of coexisting elements (Eich-Greatorex et al., 2010; Wang, Q. et al., 2016), but is also related to soil Se content and fraction (Wang et al., 2012). Activating stable Se in soil is a key process to increase the content of available Se and promote Se uptake by plants. According to the differences in water solubility and binding strength with various soil components, soil Se can be divided into different fractions, including soluble Se, exchangeable Se, iron oxide-bound Se, organic-matter-bound Se, and residual Se. These fractions are widely used to study the chemical Se forms (Wang, Q. et al., 2016; Zhang et al., 2017). Soluble Se and exchangeable Se are easily taken up by plants, and considered as plant-available fractions (Zhang et al., 2017). Iron oxide-bound Se is firmly fixed on the mineral surface, so it’s not readily absorbed by plants (Qin et al., 2012). Organic-matter-bound Se is a potential source of available Se, and it can be released into soil solution and absorbed by plant through mineralization (Kulp and Pratt, 2004; Wang et al., 2018). Some reports have indicated that low molecular weight organic acids (LMWOAs) play a key role in mobilizing Se, thus greatly increasing bioavailable Se (Qin et al., 2004). The mobilization process with LMWOAs, through competitive adsorption or desorption, can release Se from the solid phase in soil into soluble Se and exchangeable Se (Dinh et al., 2017). Li et al. (2017b) reported that organic acids can activate stable Se and increase Se availability in low pH soils.

Phosphorus (P) plays an important role in plant growth, and P deficiency limits crop production in many regions of the world (He et al., 2017b). Mainly due to the poor solubility and slow diffusion of P salts, and tight adsorption of P by iron-oxides, aluminum-oxides/hydroxides or calcium compounds in soil, P availability in soils is often very low (Turrión et al., 2018). To increase the availability of soil P, P fertilizers are commonly added to the soil to maintain crop P demand and achieve yield targets (Wang et al., 2015). The reliance on high rates of P fertilizers not only consumes the limited rock phosphate reserves, but also causes environmental pollution (Pang et al., 2018). Therefore, increasing the availability of P in soils and the amount of available P for plant uptake is very important to overcome P deficiencies in agricultural ecosystems. On the other hand, due to the strong competition between phosphate and selenite by sorption sites, it is expected to raise selenite availability to plants by the use of phosphate fertilizers (Barrow et al., 2005; Mora et al., 2008). Ngigi et al. (2019) observed that addition of P fertilizers positively affects the impact of Se fertilization with low soil P, and Li et al. (2018) got a similar conclusion in rice (*Oryza sativa*). However, some studies have conflicting results that P fertilizer has a negative effect on the uptake of Se by plants (Liu et al., 2004; Zhang et al., 2014). As yet, the effects of P fertilizers on Se uptake by plants are controversial.

Arbuscular mycorrhizal fungi (AMF) are an important component of the soil microbial community living in the rhizosphere and are present around more than 80% of terrestrial plant roots (Nanjareddy et al., 2014; He et al., 2017b). AMF are mandatory symbionts, which colonize most terrestrial plants and help in plant growth, nutrition, and tolerance for diseases (He et al., 2017c). They provide a direct link between plant roots and soil, and explore soil beyond the rhizosphere by increasing the absorptive root surface through hyphae, thus helping host plant acquire water and essential nutrients, especially P (Smith and Read, 1997; Langer et al., 2010). Due to higher phosphatase activity of the internal hyphae of mycorrhizal fungi, which can hydrolyze more organic P, mycorrhizal association benefited yield in barley by improving phosphatase activity for P uptake (Goicoechea et al., 2004). Smith and Read (2008) indicated that the inoculation of AMF can improve P uptake by increasing the volume of soil explored by roots. When roots were colonized by AMF, the mycorrhizal pathway of uptake was the dominant pathway for P acquisition (Smith et al., 2003; Watts-Williams et al., 2015). Seymour et al. (2019) also found that AMF markedly increased P content of the linseed (*Linum usitatissimum* L.). Meanwhile, there are some studies about the effects of AMF on the uptake and accumulation of Se by plants, which are controversial. In the study of Durán et al. (2016), there was no significant difference in Se concentrations between mycorrhizal and non-mycorrhizal plants. Munier-Lamy et al. (2007) reported that AMF reduced Se uptake by 30% in ryegrass. Goicoechea et al. (2015) got a similar conclusion in leaves of lettuces. Researches by Yu et al. (2011) showed that mycorrhizal inoculation increased plant uptake of P, but inhibited selenite uptake by plant roots. In contrary, Luo et al. (2019) indicated that AMF significantly enhanced Se accumulation in winter wheat in selenite-spiked soils. Nevertheless, the mechanisms related to the interaction of AMF and P input on Se uptake by alfalfa and fractions transformation in the soil systems are still not clear.

Alfalfa (*Medicago sativa* L.) is a perennial forage legume grown widely, it is sensitive to changes in soil P supply, and it is also able to accumulate Se (He et al., 2020; Peng et al., 2020). In Se-deficient areas, the production of Se-enriched alfalfa is one of the most important ways to supply Se to humans and livestock (He et al., 2019). Previous studies have reported the effects of AMF or P fertilizers on the uptake and accumulation of Se by plants, but little is known about soil available Se levels and the transformation of soil Se fractions under P and AMF interaction. The objective of this study was to investigate how AMF treatments affect the transformation of soil Se fractions in a plant-soil system with three levels of P fertilizer treatments. We hypothesized that: 1) colonization by AMF and low P availability would stimulate alfalfa roots to release more carboxylates and alkaline phosphatase; 2) AMF and P fertilizer would affect the transformation of soil Se fractions in the rhizosphere of alfalfa; 3) variations in carboxylates exudation and alkaline phosphatase activity in the rhizosphere are important for P and Se acquisition.

## Materials and Methods

### Substrate Preparation

The loessial soil was collected from the top plow layer (0–20 cm) of the Ansai County in the middle of the Loess Plateau in China (approximately 108°5’E, 36°30’N). The soil was air-dried and passed through a 2-mm sieve for the pot experiment. The texture of the soil was 45% sand, 41.6% silt, and 13.4% clay. The soil had a pH of 8.7, it had 0.1 mg kg^-1^ total Se, 0.1 g kg^-1^ total nitrogen (N), 0.5 g kg^-1^ total P, 3.3 μg g^-1^ plant-available P, 16.4 mg kg^-1^ total potassium (K), 2.8 g kg^-1^ organic matter, and 41.1 mg g^-1^ total calcium (Ca), 4.2 mg g^-1^ total magnesium (Mg), 12.4 mg g^-1^ total iron (Fe), 257 μg g^-1^ total manganese (Mn), 9.7 μg g^-1^ total copper (Cu), 37.9 μg g^-1^ total zinc (Zn). The substrates in all pots were amended with 1 mg Se kg^-1^ (supplied as sodium selenite [Na_2_SeO_3_] [Analytically pure, Xiya Reagent, China]).

The experiment included three P-application levels (0 [0P], 5 [5P], and 20 [20P] mg kg^-1^) and two mycorrhizal inoculation treatments, i.e. without mycorrhizal inoculation (−AMF) and with mycorrhizal inoculation (+AMF). The AMF inoculum specie was vesicular-arbuscular mycorrhiza *Glomus mosseae* BGC YN02 (511C0001BGCAM0022, National Infrastructure of Microbial Resources, China), supplied by the Bank of Glomeromycota in China. We cultivated each fungal isolate using capsicum (*Capsicum annuum* L) as the host species in pot cultures in a greenhouse in the Institute of Plant Nutrition and Resources, Beijing Academy of Agriculture and Forestry Sciences. The substrate of these cultures was collected after 4 months, air-dried, and controlled for the presence of viable AM fungal spores of the correct morphotype. The inoculum consisted of a mixture of soil, hyphae, spores, and infected root fragments. Non-transparent PVC tubes of 15-cm diameter and 25-cm height with a sealed bottom were used as pots for the experiment. Each pot was first filled with 4 kg of the loessial soil. The soils used for this experiment were sterilized once by autoclaving at 120°C for 2 h. The +AMF treatments contained 20 g of the mycorrhizal inoculum. For the -AMF treatments, 20 g of the same inoculum was filtered using distilled water through 11 μm filter papers (Whatman, UK) to obtain 20 ml filtrate to supply the soil with microbes without AMF communities, then the same amount of mycorrhizal inoculum was autoclaved and added to the soil in each pot.

One hundred milligrams N kg^-1^ as ammonium nitrate (NH_4_NO_3_) was used for all treatments, and 5 and 20 mg P kg^-1^ as monopotassium phosphate (KH_2_PO_4_) were used in the 5P and 20P treatment groups, respectively. In all groups, KCl was added to each pot to obtain the dose of K supply at 50 mg K kg^-1^ soil. The experiment was set as a completely randomized block design, with four replicates for each treatment. The soil in all pots was incubated for 8 weeks by watering with deionized (DI) water at about 60% field capacity in a greenhouse before growing plants.

### Plant Cultivation and Harvest

*Medicago sativa* L. cv Golden Empress, an introduced cultivar of alfalfa, was used in this study. Seeds were first sterilized in a 30% (v:v) hydrogen peroxide (H_2_O_2_) solution, rinsed with DI water repeatedly. Twenty seeds were sown in each pot and thinned to 10 plants per pot 4 weeks after sowing. DI water was added to maintain the soil moisture content at about 60% field capacity by regular weighing. Plant fresh weight (about 30 g per pot at harvest) was small compared with the water content (about 792 g per pot), therefore, it was ignored when the amount of water that should be replenished was calculated. The experiment was carried out from May 2018 to September 2018 for a total of 120 d in a greenhouse in the Institute of Soil and Water Conservation, Yangling, Shaanxi, China.

At harvest, shoots were carefully cut from the pots. The root systems were gently shaken to remove excess soil, the soil remaining attached to the roots was defined as rhizosphere soil (Pang et al., 2018). The rhizosphere soil was divided into two parts, one air-dried for analysis of Se fractions and soil P, and another stored at −20°C for the determination of alkaline phosphatase activity and microbial P immobilization.

### Collection and Determination of Rhizosphere Carboxylates

Carboxylates in the rhizosphere were extracted according to Pang et al. (2018) and He et al. (2017b). For each pot with plants, about 1.0 g fresh roots with rhizosphere soil was transferred to a beaker containing 20 ml of 0.2 mM CaCl_2_ to ensure cell integrity and gently shaken to remove the rhizosphere soil. The pH of the rhizosphere extract was measured using a pH meter. A 1 ml subsample of the rhizosphere extract was filtered through a 0.22-μm syringe filter into a 1-ml HPLC vial, then acidified with one drop of concentrated phosphoric acid, and frozen at −20°C until HPLC analysis. HPLC analysis of the elution liquid was performed using a Waters 1525 HPLC equipped with Waters 2489 detector and Alltima C-18 reverse phase column (250 × 4.6 mm, 5 μm) (Waters, Milford MA, USA). Working standards of malic acid, oxalic acid, citric acid, acetic acid, malonic acid, and tartaric acid were used to identify carboxylates at 210 nm (Cawthray, 2003). The root in the beaker after the extraction of carboxylates was cleaned, oven-dried at 60°C for 72 h, and RDM recorded. Amount of rhizosphere carboxylates were calculated as μmol g root^-1^ dry mass.

### Determination of Root Colonization by AMF

After the extraction of carboxylates, all roots in each pot were picked out and placed in individual plastic bag. These roots were washed carefully with DI water and then kept at 4°C. Fresh root subsamples were randomly taken to evaluate root AMF colonization. Roots were maintained in 10% (w/v) KOH solution in a 90°C water bath for 40 min, rinsed with water, acidified with 2% (v/v) HCl, and then stained with 0.05% (w/v) Trypan blue (Wen et al., 2019). For each root sample, 15 pieces of 1-cm segment were randomly selected and mounted on three slides for observation with a light microscope. The percentages of AM colonization were calculated under a microscope using the gridline intersect method (Giovannetti and Mosse, 1980).

### Determination of Plant Phosphorus and Selenium Concentrations

Shoots and roots were oven-dried at 60°C for 72 h, and weighed separately to obtain the dry mass. The oven-dried samples were then finely ground for analysis of P and Se concentrations. For each plant sample, about 0.5 g subsample was digested using a mixture of nitric and perchloric acid (v/v, 4:1). Plant P concentration was determined using the molybdenum blue method after digestion (Lu, 2000). For plant Se analysis, the digested solution was treated with 6 M HCl and heated for 20 min at 93–95°C to reduce all species of Se to selenite. Se concentration was determined using an atomic fluorescence spectrophotometer (AFS-230E, Beijing Haiguang Instruments Company, China) (Wang et al., 2012).

### Analysis of Soil Phosphorus and Alkaline Phosphatase Activity

Bulk soil samples were taken from each pot after being mixed thoroughly at harvest. The bulk soil was divided into two parts, one air-dried for analysis of Se fractions and soil P, and another stored at −20°C for the determination of alkaline phosphatase activity and microbial P immobilization.

Soil available phosphorus (Olsen-P) was extracted with 0.5 M NaHCO_3_, and its concentration was determined by the molybdenum blue method (Lu, 2000). Soil microbial biomass phosphorus (MBP) were determined using the method of chloroform fumigation-extraction (Brookes et al., 1985; Vance et al., 1987). Alkaline phosphatase (EC 3.1.3.1) activity was measured based on the absorption of released phenol (Guan et al., 1986).

### Determination of Soil Selenium Fractions

Soil Se fractions were determined using the sequential extraction procedure described by Wang et al. (2012). In brief, soil samples (about 1.000 g) were placed into 50 ml centrifuge tubes and extracted using different solutions (solid/liquid = 1:10) for each step. The solutions for extracting the soluble fraction (SOL-Se), exchangeable fraction (EX-Se), iron oxide-bound fraction (Fe-Se), and organic-matter-bound fraction (OR-Se) are 0.25 M KCl, 0.1 M KH_2_PO_4_-0.1 M K_2_HPO_4_, 2.5 M HCl, and 0.1 M K_2_S_2_O_8_, respectively. All solutions obtained were heated at 93–95°C for 20 min in 6 M HCl solution to transform selenate into selenite. Se concentration in the solution was determined using an atomic fluorescence spectrophotometer (AFS-230E, Beijing Haiguang Instruments Company, China) (Wang et al., 2012).

### Statistical Analysis

The data were statistically analyzed using analysis of variance (ANOVA) and Pearson correlation analysis procedures with the SPSS 20.0 statistical software. Linear regression analysis was conducted in Origin 9.0. The effect of AMF treatment (+AMF and −AMF), P fertilizer (0P, 5P, and 20P), and the interaction between the two factors were estimated with two-way ANOVA. One-way ANOVA and Tukey test were performed individually for each P fertilizer level (0P, 5P, and 20P) and AMF treatments (+AMF and −AMF). Significance level was set at 0.05. Partial least squares path modeling (PLS-PM) was used to identify the major pathways of the influences of predictor variables on plant Se uptake using the “innerplot” function of the plspm package in R 3.6.0.

## Results

### AMF Root Colonization and Plant Growth

**Figure 1:**
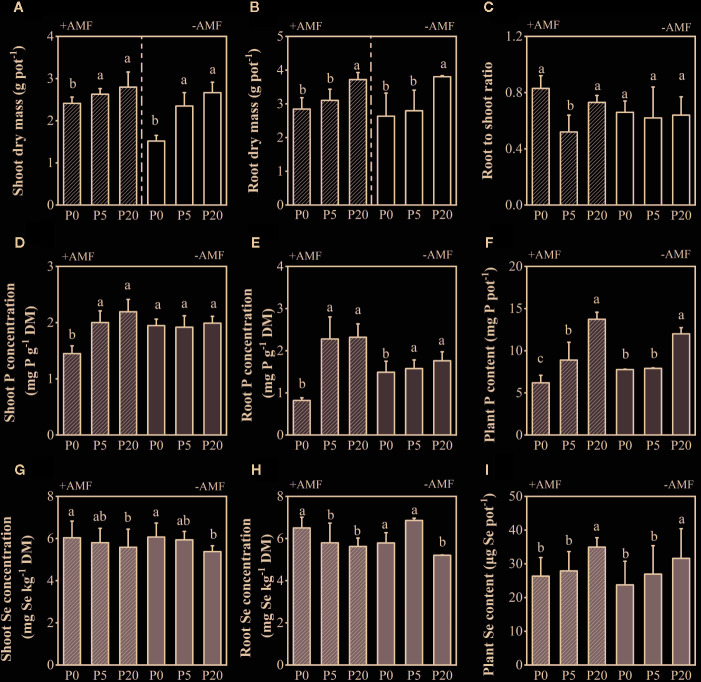
Shoot dry mass **(A)**, root dry mass **(B)**, root to shoot ratio **(C)**, P and Se concentrations **(D, E, G, H)**, total P and Se contents in plants per pot **(F, I)** for all treatments. Data are presented as means ± S.E. (*n* = 4). One-way ANOVA and Tukey’s test were performed for each AMF treatment (+AMF or −AMF). Significant differences (*P* < 0.05) between treatments are indicated with different letters above the bars.

In the +AMF treatments, the mean values of AMF root colonization rate in 0P, 5P, and 20P treatments was 54, 45, and 69% (**Figure S1**). Shoot dry mass (SDM) and root dry mass (RDM) significantly increased in 5P and 20P treatments compared to 0P treatment (*P* < 0.01), and increased in +AMF treatments compared to −AMF treatments at the same P level. The two-way ANOVA revealed that P level had a stronger effect on SDM and RDM compared to inoculation (**Table S1**). Root to shoot ratio was higher in +AMF treatments than in −AMF treatments within the 0P and 20P treatments. Inoculation and P level had a significant interaction on the root to shoot ratio (*P* < 0.01) (**Figure 1**).

### Phosphorus and Selenium Concentrations in Plants

Shoot and root P concentrations increased in 5P and 20P treatments compared to 0P treatment (**Figure 1**). Only P level had a significant effect on shoot and root P concentration, yet there was a significant interaction between P level and inoculation on shoot and root P concentrations (both *P* < 0.01). Plant P content was higher in +AMF treatment than in −AMF treatment within the 5P and 20P treatments, and it significantly increased in 5P and 20P treatments compared to 0P treatment (*P* < 0.001) (**Table S1**).

Shoot Se concentration decreased when soil P level increased, being significantly lower in 20P treatment than in 0P treatment (*P* < 0.05). Root Se concentration significantly decreased in 20P treatment compared to 0P treatment (*P* < 0.05). Only soil P level had a significant effect on shoot and root Se concentrations. Plant Se content significantly increased when soil P level increased (*P* < 0.001). At the same P level, +AMF treatment significantly increased Se content compared to −AMF treatment. (**Figure 1**; **Table S1**).

### Soil Microbial Biomass Phosphorus and Plant-Available Phosphorus

**Figure 2:**
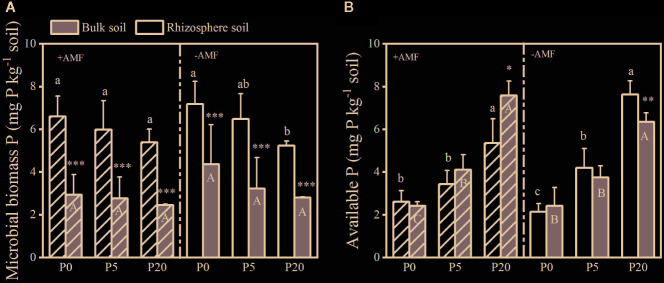
Microbial-biomass P **(A)** and available P **(B)** in the rhizosphere soil and bulk soil. Data are presented as means ± S.E. (*n* = 4). One-way ANOVA and Tukey’s test were performed in the +AMF and −AMF treatments separately for the bulk soil and rhizosphere soil. Lower-case letters indicate significant differences between the rhizosphere soil, and upper-case letters indicate significant differences between the bulk soil. One-way ANOVA significance (*P* < 0.05) to test for significant differences between the bulk soil and rhizosphere soil for each treatment is indicated by *. *P < 0.05; **P < 0.01; ***P < 0.001.

Soil MBP decreased when soil P level increased in both the rhizosphere soil and bulk soil (**Figure 2**). In all treatments, rhizosphere soil MBP was significantly higher than bulk soil MBP within the same P and inoculation treatments (*P* < 0.001). Soil Olsen-P in both the rhizosphere soil and bulk soil significantly increased with increasing P level (both *P* < 0.05). In +AMF treatment, Olsen-P was higher in bulk soil than in rhizosphere soil within the 5P and 20P treatments. On the contrary, in the −AMF treatment, Olsen-P was lower in bulk soil than rhizosphere soil within the 5P and 20P treatments. Inoculation did not have a significant effect on MBP and Olsen-P (**Table S1**).

### Soil pH, Rhizosphere Soil Alkaline Phosphatase Activity, and Carboxylates

**Figure 3:**
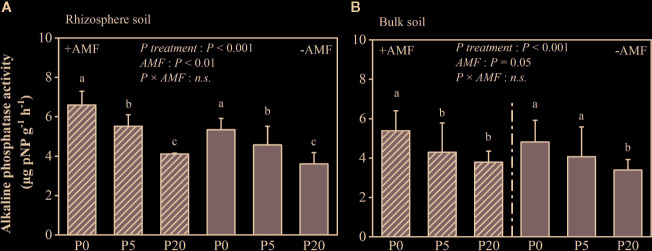
Alkaline phosphatase activity in the rhizosphere **(A)** and bulk soil **(B)**. Data are presented as means ± S.E. (*n* = 4). Two-way ANOVA and Tukey’s test were performed for each AMF treatment (+AMF and −AMF) and P treatment (P0, P5, and P20). ANOVA *P*-values are indicated in the graphs. Significant differences (*P* < 0.05) between treatments are indicated with different letters above the bars.

Rhizosphere pH was significantly lower than bulk soil pH (*P* < 0.05) and significantly declined as P level increased (*P* < 0.05) (**Figure S2**). Rhizosphere pH was 7.90–8.09 in the −AMF treatments, and 7.72–8.06 in the +AMF treatments. Alkaline phosphatase activity in the rhizosphere soil is presented in **Figure 3**. In the +AMF and −AMF treatments, alkaline phosphatase activity significantly decreased, i.e. by 16–38% (*P* < 0.001) and 14–32% (*P* < 0.001) respectively, as P level increased. Alkaline phosphatase activity was significantly higher in +AMF treatment than in −AMF treatment within the same P treatments (*P* < 0.001). According to the two-way ANOVA, AMF and P treatments did not have a significant interaction on rhizosphere alkaline phosphatase activity, and P treatment had a stronger effect compared to AMF treatment (**Table S1**). Alkaline phosphatase activity in rhizosphere soil was significantly higher than that in bulk soil (*P* < 0.05).

**Figure 4:**
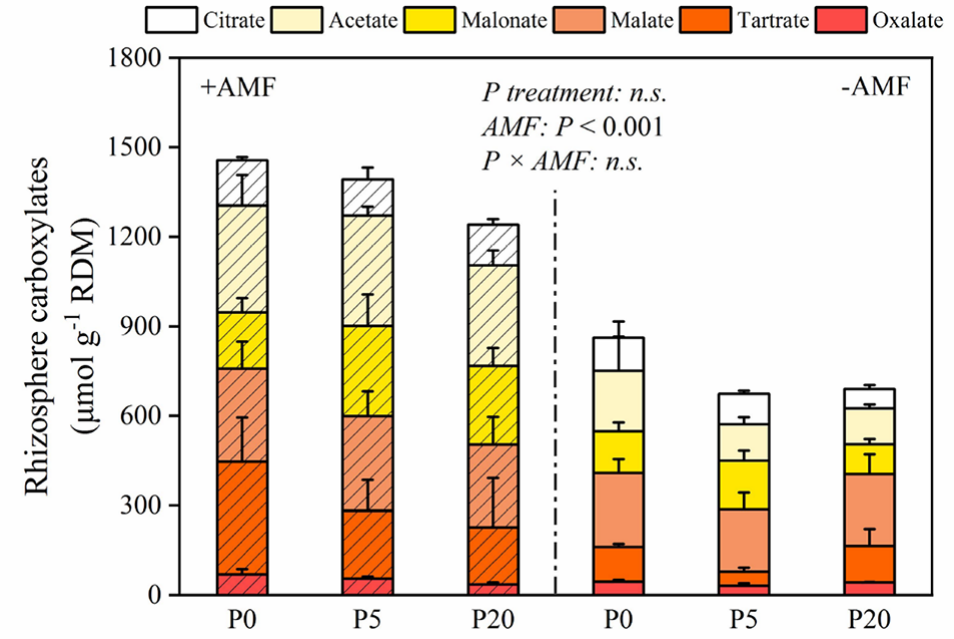
The amount of carboxylates relative to root dry mass. Data are presented as means ± S.E. (*n* = 4). Two-way ANOVA and Tukey’s test were performed for each AMF treatment (+AMF and −AMF) and P treatments (P0, P5, and P20). ANOVA *P*-values are indicated on the graph.

The amounts of carboxylates in the rhizosphere are presented in **Figure 4**. In +AMF treatment, the amount of oxalate, tartrate, malate, malonate, acetate, citrate, and the total amount of rhizosphere carboxylates measured relative to RDM was 35–69, 191–378, 278–311, 189–263, 337–369, 122–152, and 1,196–1,457 μmol g^-1^, respectively. In −AMF treatment, the amount of oxalate, tartrate, malate, malonate, acetate, citrate, and the total amount of rhizosphere carboxylates measured relative to RDM was 31–45, 47–121, 209–248, 100–163, 120–203, 65–111, and 674–863 μmol g^-1^, respectively. In both +AMF and −AMF treatments, the amounts of carboxylates decreased when soil P level increased, and it was significantly higher in +AMF treatment than in −AMF treatment within the same P treatments (*P* < 0.001) (**Table S1**).

### Soil Selenium Fractions

**Figure 5:**
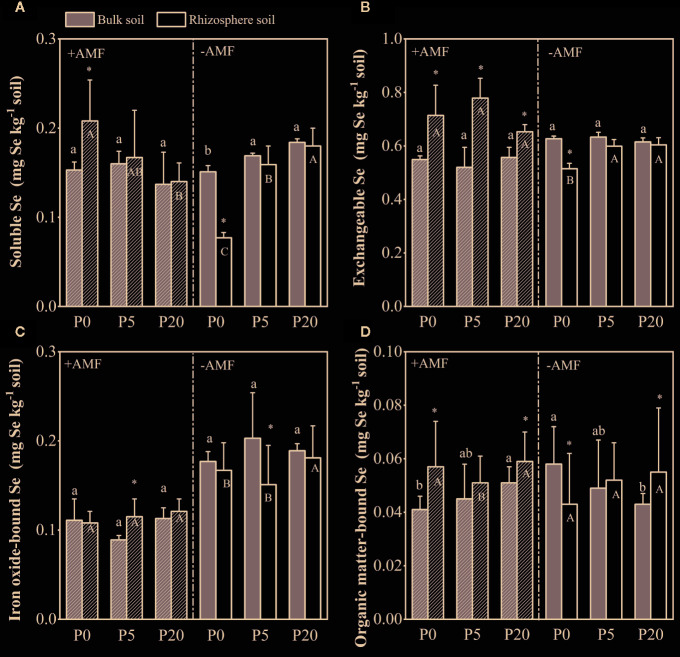
Sequential extraction results of selenium fractions in the bulk soil and rhizosphere soil. **(A)** Soluble Se; **(B)** Exchangeable Se; **(C)** Iron oxide-bound Se; **(D)** Organic matter-bound Se. Data are presented as means ± S.E. (*n* = 4). One-way ANOVA and Tukey’s test were performed for each Se fraction in the +AMF and −AMF treatments separately for the bulk soil and rhizosphere soil. Lower-case letters indicate significant differences between the bulk soil, and upper-case letters indicate significant differences between the rhizosphere soil. One-way ANOVA significance (*P* < 0.05) to test for significant differences between the bulk soil and rhizosphere soil for each treatment is indicated by *.

Se fractions in bulk soil and rhizosphere soil Se in the soil was fractionated into four fractions. The average proportions of Se in different fractions followed the order of EX-Se > SOL-Se > Fe-Se > OR-Se (**Figure S3**). In the rhizosphere soil, SOL-Se decreased in the +AMF treatment, but increased significantly in the −AMF treatment as the P level increased, while EX-Se concentration did not change significantly in the +AMF or −AMF treatment (**Figure 5**). Compared to bulk soil, SOL-Se and EX-Se concentrations in rhizosphere soil increased by 2–36 and 17–50% in the +AMF treatment, and decreased by 2–49 and 2–18% in the −AMF treatment, respectively. In the rhizosphere soil, Fe-Se concentration was higher in 20P treatment compared to 0P and 5P treatments in +AMF and −AMF treatments. In the +AMF treatment, Fe-Se concentration increased by 7–29% in rhizosphere soil compared to bulk soil, and decreased by 4–26% in the −AMF treatment. In bulk soil, OR-Se concentration increased significantly as the P level increased in +AMF treatment (*P* < 0.05), but decreased significantly in −AMF treatment (*P* < 0.05). In the rhizosphere soil, OR-Se concentration was higher in 20P treatment than in 0P and 5P treatments. In the +AMF treatment, OR-Se concentration increased by 13–39% in rhizosphere soil compared to bulk soil. No significant difference among three P levels in the bulk soil was observed in SOL-Se, EX-Se, and Fe-Se concentrations in +AMF or −AMF treatment.

### Correlations and Partial Least Squares Path Modeling

**Figure 6:**
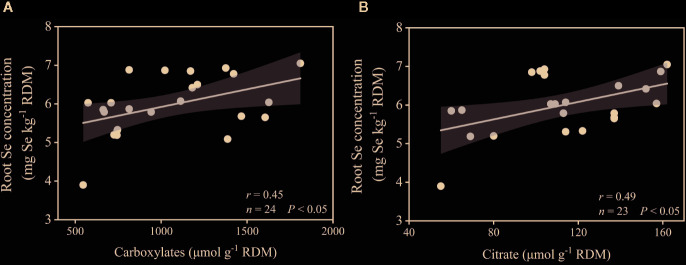
Correlation between root Se concentration and total carboxylates **(A)**, and citrate **(B)** in the rhizosphere. ANOVA *P*-values are indicated in the graphs. Gray areas are the 95% confidence intervals of the models.

Plant Se content showed a significant correlation with plant P content (*r* = 0.729, *P* < 0.01), dry mass (*r* = 0.599, *P* < 0.01), alkaline phosphatase activity (*r* = –0.391, *P* < 0.05), MBP (*r* = –0.620, *P* < 0.001), rhizosphere pH (*r* = –0.518, *P* < 0.01), Olsen-P (*r* = –0.514, *P* < 0.05), and carboxylates (*r* = –0.452, *P* < 0.05) (**Table 1**). Root Se concentration had a significant positive correlation with total carboxylates (*r* = 0.453, *P* < 0.05) and citrate (*r* = 0.485, *P* < 0.01) (**Figure 6**), but no significant correlation with oxalate (*r* = 0.045, *P >* 0.05), tartrate (*r* = 0.163, *P >* 0.05), malate (*r* = 0.014, *P >* 0.05), malonate (*r* = 0.140, *P >* 0.05), or acetate (*r* = 0.055, *P >* 0.05).

**Table 1:** Pearson’s correlation matrix for plant traits and soil properties.

	Plant P content	Plant Se content	Dry mass	Root to shoot ratio	Apase	MBP	pH	Olsen-P	Carboxylates
Plant Se content	0.729**								
Dry mass	0.588**	0.599**							
Root to shoot ratio	−0.092	0.172	0.441*						
Apase	−0.751**	−0.391*	−0.432*	0.257					
MBP	−0.556**	−0.620**	−0.638**	−0.229	0.391				
pH	−0.538**	−0.518**	−0.389	0.027	0.383	0.717**			
Olsen-P	0.736**	−0.514*	0.608**	−0.074	−0.707**	−0.540**	−0.479*		
Carboxylates	0.001	−0.452*	−0.028	0.070	0.467*	0.046	−0.049	−0.360	
AMF colonization	0.242	0.384	0.199	0.383	0.233	−0.243	−0.300	0.097	0.750**

Apase, rhizosphere alkaline phosphatase activity; MBP, microbial-biomass P; Olsen-P, available P; pH, rhizosphere pH; carboxylates, the amount of total carboxylates in the rhizosphere.

*P < 0.05. **P < 0.01; ***P < 0.001.

Correlation coefficients with P < 0.05 were shown in bolded.

**Figure 7:**
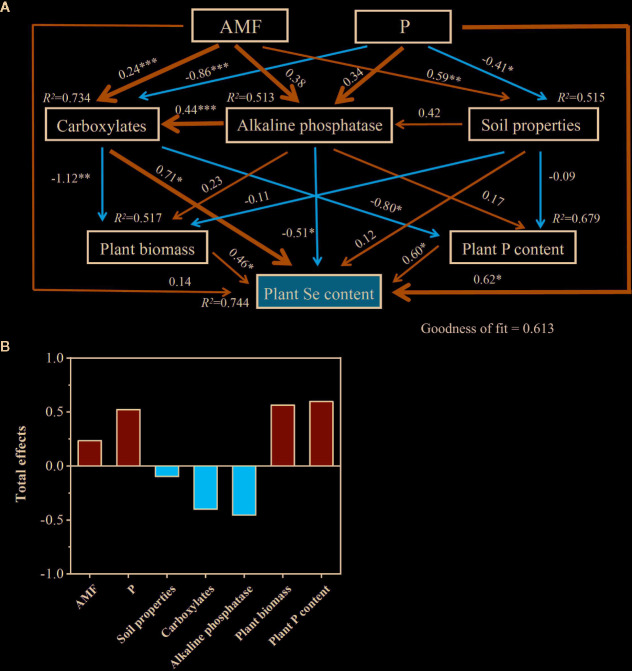
Cascading relationships of plant Se content with plant traits and soil properties. Partial least squares path modelling disentangles the major pathways of the influences of plant traits and soil properties on plant Se content **(A**, **B)**. Blue and red arrows indicate positive and negative flows of causality, respectively. *P < 0.05; **P < 0.01; ***P < 0.001. Numbers on the arrow indicate significant standardized path coefficients. *R^2^* indicates the variance of dependent variable explained by the model.

The PLS-PM identified direct and indirect effects of the colonization by AMF colonization rate, P supply rate, rhizosphere carboxylates amount, soil properties (rhizosphere pH, MBP, and Olsen-P), rhizosphere soil alkaline phosphatase activity, plant biomass, and plant P content on plant Se content (**Figure 7**). The soil properties (–0.098), rhizosphere carboxylates (–0.400), and alkaline phosphatase activity (–0.455) had negative total-effects on plant Se content, while the colonization by AMF (0.234), P supply (0.523), plant biomass (0.564), and plant P content (0.597) showed positive total-effects on it.

## Discussion

Our results show that alfalfa dry mass and shoot and root P concentrations were significantly increased by the interaction of AMF and P treatments. Patharajan and Raaman (2012) reported that alfalfa dry mass in mycorrhizal plants were significantly higher than that in non-mycorrhizal plants. Mycorrhizal symbiosis can play an important role in improving plant viability (Patharajan and Raaman, 2012; Ju et al., 2019). In the 5P and 20P treatments, inoculation of AMF increased plant P content, possibly due to the higher plant growth and greater mobilization of soil P by external hyphae (Asghari et al., 2005; Parihar et al., 2019). Some previous reports also indicated that mycorrhizal colonization can improve the P status of plants (Chen et al., 2010; Goicoechea et al., 2014; He et al., 2017c). The result that mycorrhizal colonization rate was the highest in P20 treatment in the present study did not agree with the conclusions of some studies, which have shown a negative correlation between AMF colonization rate and soil available P (Asghari et al., 2005; Kowalska and Konieczny, 2019). It might be due to the relatively low levels of available P in the soil in all treatments (<10 mg available P kg^-1^ soil). Fornara et al. (2019) reported that when soil available P concentration reached 50 mg kg^-1^ and beyond, AMF colonization rate decreased significantly. On the other hand, AM fungi may be parasitic to their host plants if the net cost of AM symbiosis exceeds the net benefit (Johnson et al., 1997). AM symbiosis could be altered by the level of soil available P, and mutualism likely occur in P-deficient soils (Johnson et al., 2015). In the results of Liu et al. (2020), AMF colonization rate was the highest in P30 treatment compared with the control and P100 treatments. In the present study, both AMF colonization rate and P content were the highest in P20 treatment, likely indicating the best benefit of AM symbiosis (Sawers et al., 2017).

In our results, shoot Se concentration decreased from 6.0 to 5.5 μg kg^-1^, but plant Se content per pot increased from 26 to 35 μg as the P level increased in the +AMF treatments. It can be explained by the dilution effect that the increased biomass diluted the Se concentration in plants (Mora et al., 2008; Lee et al., 2011). Carter et al. (1972) found that P-fertilizer application increased Se content in alfalfa grown in alkaline soils, and speculated that the addition of P replaced soil-adsorbed Se, since P had a higher adsorption capacity than Se. The presence of P in soil decreased Se adsorption on soil surfaces, and increased Se concentration in soil solution, thereby increasing Se uptake by plants (Nakamaru and KenjiSekine, 2008; Lee et al., 2011). The effects of AMF on plant Se uptake are still not clear. In the study of Yu et al. (2011), mycorrhizal inoculation inhibited Se uptake by plant roots, due to enhanced binding of Se on hyphae and root surface, which inhibited further movement of Se to roots. However, Luo et al. (2019) reported that mycorrhizal inoculation significantly increased uptake of selenate and selenite by increasing the valid absorption area of roots in winter wheat (*Triticum aestivum* L.). Golubkina et al. (2019) reported that colonization by AMF increased yield and Se content of shallot bulbs (*Allium cepa* L.) by increasing the antioxidant activity of ascorbic acid when compared with the non-inoculated control. Most of previous studies have been focused on the effects of only P input or mycorrhiza colonization on plant Se uptake. Our study demonstrated for the first time that the synergistic effect of colonization by AMF and low P input promoted Se uptake by alfalfa, and the release of carboxylates, especial citrate, and alkaline phosphatase in the rhizosphere was the key factor driving increased Se uptake. In the present results, the AMF had a positive effect on plant Se content, which was increased by 5–10% with AMF. Our hypothesis that carboxylates are important for Se acquisition was supported by the observation of a significant positive correlation between root Se concentration and total carboxylates (*r* = 0.45, *P* < 0.05), especially citrate (*r* = 0.49, *P* < 0.01). There are studies showing that root exudates can compete with Se for sorption sites and reduce the retention of Se in soil, thus promoting the release of Se and facilitating plant Se uptake (Kaiser and Guggenberger, 2003; Adeleke et al., 2017; Dinh et al., 2017). Furthermore, the results of PLS-PM also supported that the colonization by AMF directly determined the carboxylates in the rhizosphere, and indirectly caused the increase of plant Se content.

Inoculation by AMF can affect plant rooting patterns as well as the supply of available nutrients to hosts, thereby changing the composition and quantity of root exudates, which may modify fungal and microbial activity (Barea et al., 2005). Lower P availability and +AMF stimulated plant roots to release more carboxylates to the soil, and AMF had a stronger effect than P treatments on the carboxylates content (**Table S1**). The amount of total carboxylates had a positive correlation with root to shoot ratio, alkaline phosphatase activity, and MBP (**Table 1**). Given the high alkaline phosphatase activity close to the roots, alfalfa roots apparently released substantial amounts of carboxylates. Roots inoculated by AMF can improve the acquisition of the mobilized P (Gerke, 2015). The release of carboxylates, especially oxalate and citrate, was considered the most effective way of P mobilization (Gerke, 2015). Compared to −AMF treatment, oxalate and citrate concentrations significantly increased in +AMF treatment within the same P levels. The increase in microbial activity and the change in microbial community structure could have affected the oxalate and citrate secretion (Dessaux et al., 2016; Bornø et al., 2018).

Soil phosphatases affect soil P cycling and can be affected by many factors, including rhizosphere processes and growth periods (Ye et al., 2018; Duan et al., 2019). Some reports have indicated that the high activities of the rhizosphere enzymes are due to their release by roots or fungi (Asmar et al., 1995; Kandeler et al., 2002; Wang, Y. L. et al., 2016). Our results showed that alkaline phosphatase activity was significantly affected by the P and AMF treatments. Within the same AMF and P treatments, alkaline phosphatase activity was significantly higher in rhizosphere soil than in bulk soil. We also found that alkaline phosphatase activity had a positive correlation with root to shoot ratio (**Table 1**). Therefore, the increase in alkaline phosphatase activity in rhizosphere soil might be due to release greater amounts of alkaline phosphatase from the roots. Wang, Y. L. et al. (2016) got similar results in canola (*B. napus* cv. MARIE). Kandeler et al. (2002) also revealed that alkaline phosphatase activity was affected by the presence of maize roots. Furthermore, inoculation by AMF can influence soil microbial community, which determined the potential for enzyme synthesis (Kandeler et al., 2002). In our study, alkaline phosphatase activity in the rhizosphere was higher in +AMF treatment compared with −AMF treatment, and had a significant positive correlation with carboxylates and MBP. The differences between +AMF and −AMF treatments in alkaline phosphatase activity can be explained by the increased microbial biomass (Li et al., 2017a) and the shift in microbial community structure (Ventura et al., 2014).

Se availability depends not only on the content of Se but also on its fractions in the environment (Dinh et al., 2017). Some reports indicated that SOL-Se and EX-Se are available for plant uptake because they are free in soil solution or weakly adsorbed on the surface of soil particles (Kulp and Pratt, 2004; Wang, Q. et al., 2016). In this study, the main Se fraction in the soil was EX-Se. EX-Se concentration was significantly higher in +AMF treatment than that in −AMF treatment, and higher in the rhizosphere soil than that in bulk soil. EX-Se had a positive correlation with the amount of carboxylates (**Table S2**). Previous reports indicated that organic acids significantly reduced the number of sorption sites by affecting soil surface characteristics (Kaiser and Guggenberger, 2003; Zhu et al., 2016). Kaiser and Guggenberger (2003) indicated that after equilibration for 24 h, surface sorption sites were reduced by 33.8% by oxalate. Therefore, carboxylates compete with Se for adsorption sites, thereby reducing the retention of Se in soil and promoting the release of soil Se, promoting plant Se uptake (Adeleke et al., 2017; Dinh et al., 2017). On the other hand, EX-Se and SOL-Se concentrations significantly increased as P supply increased. It also can explain the increased plant Se content by the increasing available Se concentrations. Meanwhile, compared to −AMF treatments, Fe-Se concentrations were decreased by 23–35%, and the total amount of carboxylates were increased by 41–53% in +AMF treatments. Fe-Se is firmly fixed by mineral surfaces and thus not readily absorbed by plants. This process is controlled by the quantity and quality of organic acids (Qin et al., 2012). Our results indicated that the increased amounts of organic acids caused the transformation of stable Se into available Se forms that are relatively available to plants.

## Conclusion

The present study provides an integrated perspective of how AMF and P fertilizer enhance the Se acquisition by alfalfa *via* altering the rhizosphere. Colonization by AMF and low P availability stimulated alfalfa roots to release more carboxylates and alkaline phosphatase, which are important in P and Se acquisition. Rhizosphere carboxylates, especially citrate, increased when roots were colonized by AMF and under low P input, indicating that the response of alfalfa to AMF and P fertilizer can alter the composition and amounts of root exudates. The presence of P in soil reduced Se adsorption on soil surfaces, and increased Se concentration in soil solution, thereby increasing Se uptake by alfalfa. Furthermore, the increased amounts of carboxylates likely reduced the retention of Se in soil, caused the transformation of stable Se into available Se forms and consequently promoted the absorption of Se by alfalfa. Therefore, the variations in carboxylates exudation and alkaline phosphatase activity in the rhizosphere might play a major role in promoting P and Se acquisition by alfalfa. The results in this study are valuable for understanding Se and P uptake by alfalfa and the transformation of Se fractions in soil under the interaction between AMF and P fertilizers.

## Data Availability Statement

The datasets generated for this study are available on request to the corresponding authors.

## Author Contributions

HH conceived and designed the experiments. All authors performed the experiments. QP was responsible for preparing the first draft of the manuscript, and HH and XZ revised the manuscript. All authors contributed to the article and approved the submitted version.

## Funding

This work was financially supported by The National Key Research and Development Plan of China (2017YFC0504504), The National Natural Science Foundation of China (41301570), The Light of West China Program of Chinese Academy of Sciences, and Fundamental Research Funds for Central Universities in China.

## Conflict of Interest

The authors declare that the research was conducted in the absence of any commercial or financial relationships that could be construed as a potential conflict of interest.
