# Supplementary material for: The Interaction of Arbuscular Mycorrhizal Fungi and Phosphorus Inputs on Selenium Uptake by Alfalfa (Medicago sativa L.) and Selenium Fraction Transformation in Soil
Source: Front Plant Sci. 2020 Jun 26;11:966. doi: 10.3389/fpls.2020.00966 (PMC7333729; doi:10.3389/fpls.2020.00966)
Supplement: Supplementary file 1 [file DataSheet_1.doc]

**Table S1** Two-way ANOVA significance for plant traits and soil parameters with factors of AMF treatment (+AMF and -AMF) and P level (0P, 5P, and 20P).

| Alfalfa dry mass and P and Se | AMF | P level (P) | AMF×P |
| --- | --- | --- | --- |
| Shoot P concentration (mg g^-1^) | n.s. | ** | ** |
| Root P concentration (mg g^-1^) | n.s. | *** | ** |
| Plant P content (mg pot^-1^) | n.s. | *** | * |
| Shoot Se concentration (mg kg^-1^) | n.s. | * | n.s. |
| Root Se concentration (mg kg^-1^) | n.s. | * | n.s. |
| Plant Se content (μg pot^-1^) | n.s. | *** | n.s. |
| Shoot dry mass (g pot^-1^) | * | ** | n.s. |
| Root dry mass (g pot^-1^) | n.s. | ** | n.s. |
| Root to shoot ratio | n.s. | *** | ** |
| Rhizosphere carboxylates |  |  |  |
| Oxalate | ** | ** | ** |
| Tartrate | *** | n.s. | n.s. |
| Malate | * | n.s. | n.s. |
| Malonate | *** | * | n.s. |
| Acetate | *** | n.s. | n.s. |
| Citrate | *** | ** | * |
| Total carboxylates | *** | n.s. | n.s. |
| Rhizosphere soil properties |  |  |  |
| MBP | n.s. | * | n.s. |
| Olsen-P | * | *** | * |
| Alkaline phosphatase activity | ** | *** | n.s. |
| pH | n.s. | ** | n.s. |
| Bulk soil properties |  |  |  |
| MBP | n.s. | n.s. | n.s. |
| Olsen-P | n.s. | *** | * |
| Alkaline phosphatase activity | n.s. | ** | n.s. |
| pH | ** | *** | ** |

*, *P* < 0.05; **, *P* < 0.01; ***, *P* < 0.001.

**Table S2** Pearson’s correlation matrix for selenium fractions and carboxylates in the rhizosphere.

|  | SOL-Se | EX-Se | Fe-Se | OR-Se |
| --- | --- | --- | --- | --- |
| Carboxylates | 0.037 | **0.526*** | **-0.711**** | -0.081 |
| Oxalate | 0.205 | 0.345 | **-0.479*** | 0.058 |
| Tartrate | 0.244 | 0.338 | **-0.516**** | 0.105 |
| Malate | 0.078 | 0.317 | **-0.587**** | 0.036 |
| Malonate | 0.001 | **0.559**** | **-0.514*** | -0.102 |
| Acetate | 0.069 | 0.331 | **-0.631**** | -0.124 |
| Citrate | -0.168 | 0.231 | **-0.692**** | 0.061 |

Abbreviations: SOL-Se, soluble Se; EX-Se, exchangeable Se; Fe-Se, iron oxide-bound Se; OR-Se, organic matter-bound Se. *, *P* < 0.05. **, *P* < 0.01; ***, *P* < 0.001.

**FIGURE CAPTIONS:**

**Figure S1.** Mycorrhizal colonization rate of alfalfa fine roots with arbuscular mycorrhizal fungi (AMF).

**Figure S2.** The pH in the bulk soil and rhizosphere soil in all treatments.

**Figure S3.** The proportion of different selenium fractions in the bulk soil (a) and rhizosphere soil (b).





**Figure S1.** Mycorrhizal colonization rate of alfalfa fine roots with arbuscular mycorrhizal fungi (AMF). Data are presented as means ± S.E. (*n* = 4). One-way ANOVA and Tukey’s test were performed for three P treatments. Significant differences (*P* < 0.05) between treatments are indicated with different letters above the bars.





**Figure S2.** The pH in the bulk soil and rhizosphere soil in all treatments. Data are presented as means ± S.E. (*n* = 4). One-way ANOVA and Tukey’s test were performed for each treatment (+AMF Rhizosphere, +AMF Bulk, and -AMF Rhizosphere, -AMF Bulk). ANOVA *P*-values are indicated in the graph.





**Figure S3.** The proportion of different selenium fractions in the bulk soil (a) and rhizosphere soil (b). Data are presented as the means of four replicates. Values were expressed as the percentage of Se concentration in each fraction to the sum of Se concentration in bulk soil and rhizophere soil.
